# Full-field quantitative phase and polarisation-resolved imaging through an optical fibre bundle

**DOI:** 10.1364/OE.27.023929

**Published:** 2019-08-06

**Authors:** George S. D. Gordon, James Joseph, Travis Sawyer, Alexander J. Macfaden, Calum Williams, Timothy D. Wilkinson, Sarah E. Bohndiek

**Affiliations:** 1 Now at: Department of Electrical and Electronic Engineering, The University of Nottingham, University Park, Nottingham, NG7 2RD, UK; 2 Department of Engineering, University of Cambridge, 9 JJ Thomson Avenue, Cambridge, CB3 0FA, UK; 3 Department of Physics, Cavendish Laboratory, JJ Thomson Avenue, Cambridge, CB3 0HE, UK; 4 Cancer Research UK Cambridge Institute, Li Ka Shing Centre, Robinson Way, Cambridge, CB2 0RE, UK; 5 tdw13@cam.ac.uk; 6 seb53@cam.ac.uk

## Abstract

Flexible optical fibres, used in conventional medical endoscopy and industrial inspection, scramble phase and polarisation information, restricting users to amplitude-only imaging. Here, we exploit the near-diagonality of the multi-core fibre (MCF) transmission matrix in a parallelised fibre characterisation architecture, enabling accurate imaging of quantitative phase (error <0.3 rad) and polarisation-resolved (errors <10%) properties. We first demonstrate accurate recovery of optical amplitude and phase in two polarisations through the MCF by measuring and inverting the transmission matrix, and then present a robust Bayesian inference approach to resolving 5 polarimetric properties of samples. Our method produces high-resolution (9.0±2.6μm amplitude, phase; 36.0±10.4μm polarimetric) full-field images at working distances up to 1mm over a field-of-view up to 750×750*μ*m^2^ using an MCF with potential for flexible operation. We demonstrate the potential of using quantitative phase for computational image focusing and polarisation-resolved properties in imaging birefringence.

## 1. Introduction

Imaging through optical fibres is performed in a range of applications, including biomedical endoscopy and industrial inspection, which exploit their small diameter (typically <1mm) and high flexibility. Unfortunately, optical fibres inherently scramble phase and polarisation information due to bending- and temperature-induced variations in glass refractive index, limiting these applications to amplitude-only imaging. This means that the wealth of information available from light phase and polarisation, relating for example to local scattering and birefringence, is lost [1–3]. Measured phase and polarisation could, however, be unscrambled by measuring and inverting the fibre transmission matrix (TM); a complex linear mapping between the two fibre facets [4].

Single-mode fibres (SMFs) with distal scanning [5], multi-mode fibres (MMFs) [6–8], and fused multi-core fibres (MCF) comprising many thousands of light-guiding cores [9] have all been investigated for imaging applications. In terms of recovery of information beyond optical amplitude, SMFs only record point measurements so are unsuitable for recovering the relative phase between points on a wavefront without a very high degree of laser and sample stability [10,11]. While MMF TMs can in theory be predicted *a priori* [12], in reality, frequent *in situ* TM characterisation is necessary to account for thermal fluctuations and motion of the fibre, which represents a significant challenge for longitudinal imaging during an inspection procedure. Several recently reported designs use phase and polarisation information to recover MMF amplitude-only images [7,13], but do quantitatively determine phase or polarisation images. Deep-learning approaches have been successful in recovering both amplitude and phase images via MMF, but polarimetric imaging has not yet been demonstrated [14]. MCFs have a near-diagonal TM resulting in minimal amplitude scrambling, hence are already employed in a range of biomedical endoscopy indications [15]. MCFs have also been used to record interferometric images in a biomedical context [16], however, the highly scrambling nature of MCF has limited such methods to imaging differences in phase between a reference image and an image of mechanically perturbed tissue.

Previous investigations using TM characterisation in conventional non-sparse Hadamard [17] and Fourier bases [18] in MCFs have been limited to wavefront shaping and wide-field imaging of a single polarisation. By contrast, here we exploit the near-diagonality of the MCF TM to enable a novel parallelised *in situ* fibre characterisation that could be applied during flexible fibre imaging and is scalable to higher resolution MCFs without additional experimental overhead. Our holographic fibrescope produces full-field images of amplitude and quantitative phase in two polarisations. Relying on phase retrieval, it achieves this simply with a low-cost laser diode and one camera, comparing favourably to systems requiring costly solid-state lasers [12,19] or 2-4 cameras [12], although an additional spatial light modulator (SLM) is required.

These raw measured optical fields are used as input to a robust Bayesian inference algorithm that resolves 5 polarimetric parameters. Bayesian inference is known to be more robust to noise than clustering or pseudo-inverse approaches for polarimetric imaging [20,21]. Here, we further exploit Bayesian inference to jointly estimate posterior probabilities of neighbouring pixels, which is essential for compensating beam misalignment.

Here, we first demonstrate recovery of amplitude and phase images in two polarisations using a novel parallelised transmission matrix approach. We then show that quantitative phase recovery enables computational refocusing [6]. Finally, we demonstrate polarimetric imaging of birefringent and diattenuating samples. Our results indicate that the proposed parallelised fibre characterisation architecture can be used to extract quantitative phase and polarisation-resolved properties from imaging data at high resolution and with high accuracy.

## 2. Methods

### 2.1. Optical set-up

**Fig. 1.**
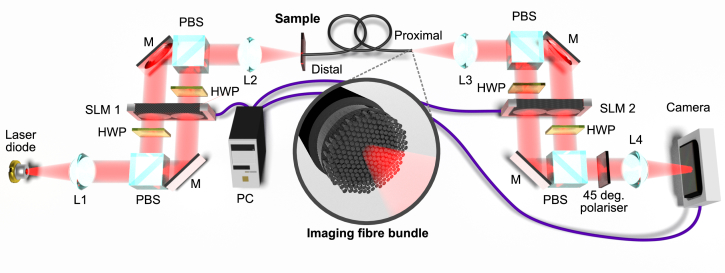
Overview of optical design for transmission matrix characterisation and recovery of phase and polarisation images. Liquid-crystal SLMs are combined with polarising beam splitters and waveplates to perform polarisation-diverse digital holographic imaging through an MCF. SLM1 illuminates the sample with the desired optical field and enables fibre characterisation, while SLM2 enables imaging of amplitude, phase and polarisation of light emerging from the fibre bundle. Abbreviations: spatial light modulator, SLM; polarising beam splitter, PBS; half wave plate, HWP; lens, L; mirror M.

A schematic overview of the optical set-up configured to achieve dual-polarisation operation [22,23] is shown in Fig. 1

. Illumination is provided by a laser diode operating at 852 nm with a power output of 35 mW (DBR852S, Thorlabs). This wavelength is optimal for our MCF (FIGH-06-350G, Fujikura; 2m length, 6000 cores, core diameter ∼2.9μm, core spacing 4.4*μ*m, outer diameter 350±20μm) because inter-core coupling is low enough to preserve the approximate image shape while still ensuring predominantly single-mode light guiding in each core. This not only enables relatively easy alignment, but is also ensures a near-diagonal structure, which is critical for the parallelised TM characterisation method.

We expect our approach to work for longer fibres because it depends fundamentally on accurate TM reconstruction which, due to the very low-losses of commercial optical fibres (<0.2 dB/km at 1550nm), has been demonstrated in mode-division multiplexing at lengths of over 2km [24] and imaging over lengths up to 1km [14]. However, longer fibres experience increased mode-coupling making the TM less diagonal thereby reducing the extent to which sparsity can be exploited for parallelised characterisation.

Spatial light modulators (SLMs; 1920 × 1080 resolution, PLUTO-NIR-015, Holoeye) are used for control of both illumination (SLM1) and detection (SLM2). One SLM characterises the fibre and illuminates the sample while the other performs phase shifting interferometry between polarisations for imaging. The laser diode output is split into two orthogonally polarised components using a polarising beam splitter (PBS; PBS102, Thorlabs) and the 2:1 aspect ratio of the SLM is then exploited, enabling each half of SLM1 to control one of the two orthogonal polarisations. The nematic liquid crystal on silicon (LCoS) SLM is used in phase-modulation mode to introduce a programmable phase shift (0 to 2*π*) at each pixel. This requires horizontally polarised incident light. Half wave plates (WPF-2-12.7x12.7-H-850, FOCtek Photonics) are therefore used in one arm to convert vertically polarised incident light to the required horizontal polarisation, and in the other arm to restore horizontally polarised reflected light to its original vertical polarisation. The two arms are recombined via a second PBS. Our illumination path design provides the ability to perform independent spatial modulation and phase shifting of polarisation states to create arbitrary polarisation wavefronts.

Samples are mounted in an automated stage (NanoMAX TS XYZ, Melles-Griot). Samples larger than the fibrescope field of view (FOV) were translated in front of the distal facet of the MCF with a fixed ∼15% overlap between positions, which enabled stitching and phase matching of adjacent images. Light passing through the sample is collected by the distal end of the MCF. The resulting FOV is approximately 200×200μm^2^ at 0 mm working distance to the sample and typical imaging operation uses illumination power of 3.2 mWcm^−2^ in the sample plane.

Having traversed the MCF, the imaging path is again split into two arms via a PBS (PBS102, Thorlabs), with each half of SLM2 modulating one arm. These arms are recombined and passed through a linear polariser oriented at 45° to the axes of the polarised components. The light is finally detected at the camera (4.2 megapixel, 90 fps MQ042RG-CM; Ximea) where the amplitude is recorded. One half of SLM2 is used to display a series of 7 parabolic phase masks, affecting one polarisation arm, and the other half is set to divert light away from the camera, effectively disabling the other arm. This results in a sequence of defocussed images of the fibre facet, termed a ‘through-focus stack’. A phase retrieval algorithm processes the stack to recover the phase in the corresponding polarisation of the optical field at the camera [25]. This approach rapidly converges on an appropriate phase profile and is known to be robust in the presence of noise. To determine the full polarisation state, the other half of SLM2 is then activated and phase-shifting interferometry between the two polarisation states is performed to determine their amplitude and phase relationship [23,26]. The 45° polariser between SLM2 and the camera results in coupling of the horizontal and vertical polarisations into a common basis, allowing interference. In total, 11 camera frames are required to recover a full amplitude, phase and polarisation image (7 for phase retrieval in one polarisation, 4 for phase-shift interferometry between polarisations). In this way, an image of the amplitude and phase in two polarisations of the optical field is recorded.

Conventionally, phase imaging is achieved by having a reference arm from the laser source interfere at the detector with the field from the sample arm. However, this requires long-coherence length lasers (or else very precise path-length matching) and is highly sensitive to perturbations over time [7,19]. By contrast, our phase-retrieval approach needs only interference of two orthogonal polarisations via a 45° polariser in front of the camera (Fig. 1), meaning the minimum required coherence length depends only on the fibre’s birefringence. Typical bending-induced birefringence for a 2m fibre may introduce a differential optical thickness of ∼1−5μm [27], with highly birefringent fibres introducing ∼100μm [28,29]. Therefore, the coherence length of the laser may be as low as ∼100μm, easily achievable using low-cost laser diodes; the device here has a coherence length of 1m. Further, removing the need to match the length of and stabilise an external reference arm offers a significant practical advantage in constructing the experiment.

The SLMs and camera are synchronised via a GPU accelerated PC and controlled using custom software written in C/C++. Unlike with multimode fibres, where the input field at the distal fibre facet must be imaged with a second camera during calibration [7], using an imaging MCF preserves the approximate form of the images so the input field can be reconstructed from a single camera image when combined with accurate simulations of the holograms displayed on SLM1.

### 2.2. System operation

**Fig. 2.**
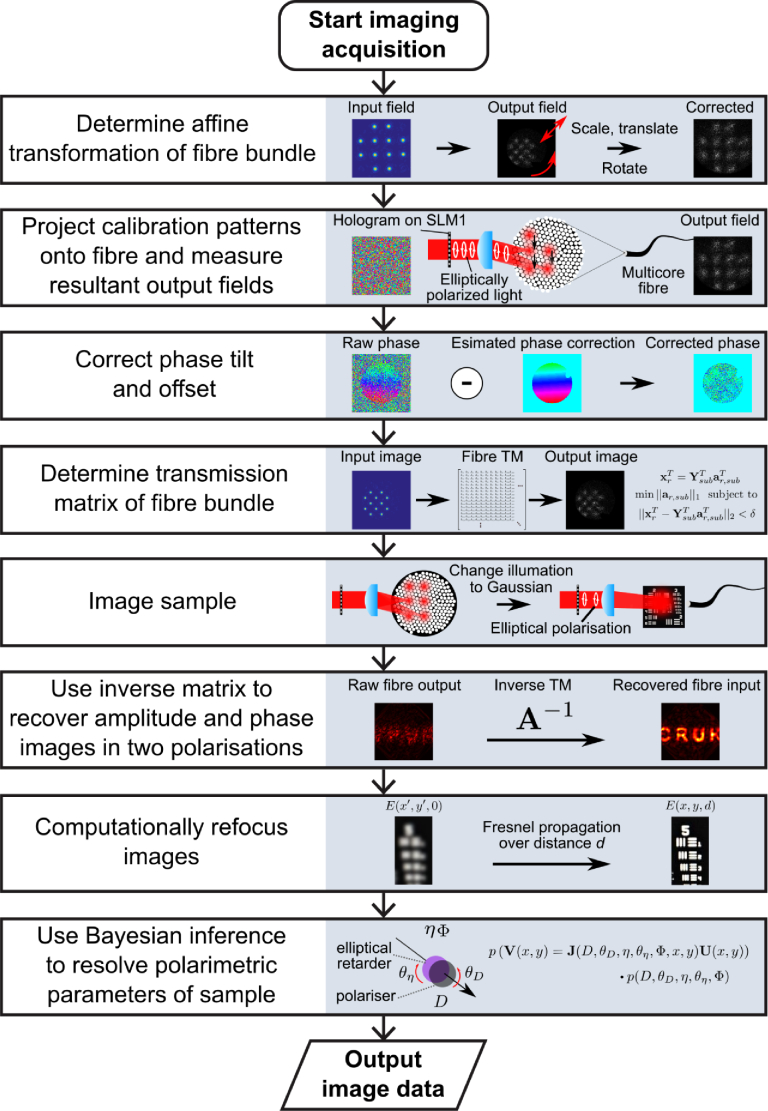
Overview of imaging operation. First, the translation, rotation and scaling of the proximal facet as it appears on the camera (relative to SLM1) is determined. Then, TM calibration patterns are projected onto the fibre and the output fields recorded. These output fields must be then corrected to compensate for phase drift [26]. Next, by comparing simulated calibration patterns with measured outputs, and splitting them up to exploit parallelisation, the TM of the MCF is reconstructed. An identity matrix represents a perfect image-preserving fibre, while variation of diagonal elements and the presence of non-zero off-diagonal elements represent varying degrees of scrambling. Next, the sample is imaged by illumination with a broad Gaussian beam in several elliptical polarisation states. The inverse of the determined TM is then multiplied by the raw measured field to reconstruct the amplitude and phase in two polarisation of a sample placed at the distal facet. After then correcting for image defocus using Fresnel propagation, Bayesian inference is performed on the recovered sample fields over different elliptical illumination states in order to resolve polarimetric properties.

The overview of the system operation procedure is shown in Fig. 2

. Before generating holograms for TM determination and sample illumination, two calibration steps must be performed: aberration correction, to correct for curvature of the SLM surface; and orientation correction, so the image of the MCF on the camera is corrected relative to the actual orientation of the distal facet of the MCF.

The well-established formalism of a transmission matrix as a discretisation of linear propagation integrals is used here [30]. We consider a sampled and vectorised representation of the optical field at the distal facet of the fibre, x, to be related to a sampled and vectorised optical field at the proximal facet, y by the transmission matrix, A as:

(1) y=Ax

**Fig. 3.**
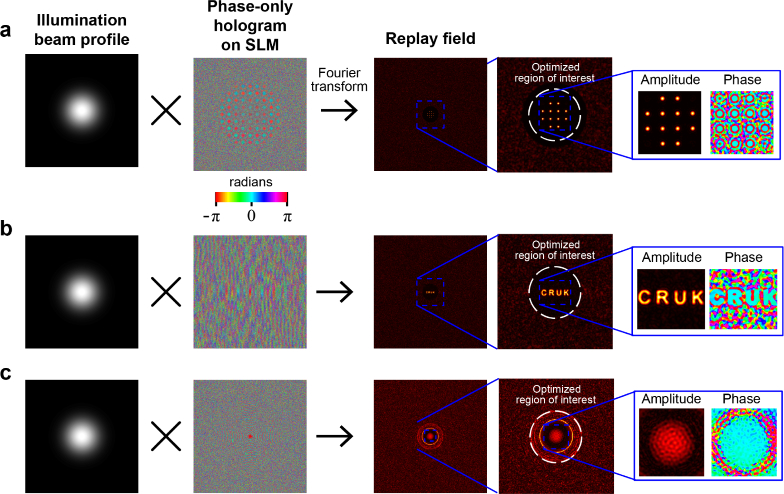
Holograms used for transmission matrix determination, verification, and sample illumination. a) An array of 12 equispaced spots are used to parallelise the fibre characterisation process. Each spot is designed to have an approximately Gaussian profile, however due to the rectangular aperture imposed by the finite extent of the spatial light modulator, in reality they are more like sinc functions with attenuated side lobes. b) Text logo used for verifying reconstruction algorithm. c) Broad, Gaussian amplitude, flat phase illumination profile used for imaging samples.

For both TM characterisation and sample illumination, the field **x** is controlled by displaying an appropriate hologram on SLM1. Three distinct holograms are used for operation of the fibrescope. The first is an array of 12 equispaced spots (Fig. 3(a)

) used for parallelised characterisation of the MCF TM before the introduction of the sample. The choice of 12 spots ensures individual spots are spaced sufficiently far from each other that there is negligible interference between them at the proximal facet of the MCF. Using an array of spots physically parallelises the characterisation process. The second hologram provides a test pattern that can be used to confirm the accuracy of the TM result. The short text phrase (CRUK) provides a useful pattern for this purpose (Fig. 3b). The third hologram, used for sample imaging, should ideally provide a uniform illumination with a flat phase front (Fig. 3c). However, since this is passed through the same lens that is used to produce the array of spots, in reality the amplitude profile converges to a Gaussian profile while the phase profile is flat to within ± 0.15 rad. To achieve high fidelity within the replay field region of interest (ROI), it is often necessary for the hologram to direct much of the light to regions outside of the ROI, meaning that the ratio of light power within the ROI to the total in the replay field may be as low as 1.7% during sample imaging.

To compute the holograms, the illumination profile incident on the SLM (a collimated laser beam) is multiplied by a pixellated phase profile to be displayed on the SLM and the Fourier transform is taken to establish the image expected at the focus of the second lens in the replay field (Fig. 3). The pixellated phase profile, or hologram, is then optimised to produce the desired replay field. As the hologram is phase-only, light can only be redistributed, not attenuated, which limits the range of possible replay fields for a given illumination. Therefore, to generate arbitrary patterns for a given illumination, a region of interest (ROI) is defined to restrict the part of the replay field that is constrained during the optimisation. Optimisation is then achieved by minimising the difference between the ROI in the replay field and the target field using a simulated annealing algorithm to find a globally optimal hologram [31]

### 2.3. Transmission matrix measurement

The calibration measurements required for TM recovery are achieved by translating the array of 12 spots (Fig. 3(a)) through 81 positions (over a 9×9 grid) in 9*μ*m steps (limiting spatial resolution). At each position, the spots are projected in 3 distinct elliptical polarisation states, resulting in a total of 81×3=243 inputs. The choice of a 9 × 9 grid size ensures that about half the cores in either dimension inside the imaging area will be characterised. This reduces the TM size by a factor of 4 for faster processing. A minimum of two polarisation states are required to resolve the 2 orthogonal polarisation components but further states can reduce noise. Using 3 states produces a ∼ 33% reduction in noise, resulting in visibly improved imaging performance. Using 4 states produces a further but smaller differential improvement. We choose 3 states as an empirically acceptable trade-off between minimising noise and minimising experimental acquisition time. Elliptical states are used so as to maximise power transmitted through the fibre, thereby minimising the effect of noise compared to linear states with only half the power.

### 2.4. Transmission matrix recovery

**Fig. 4.**
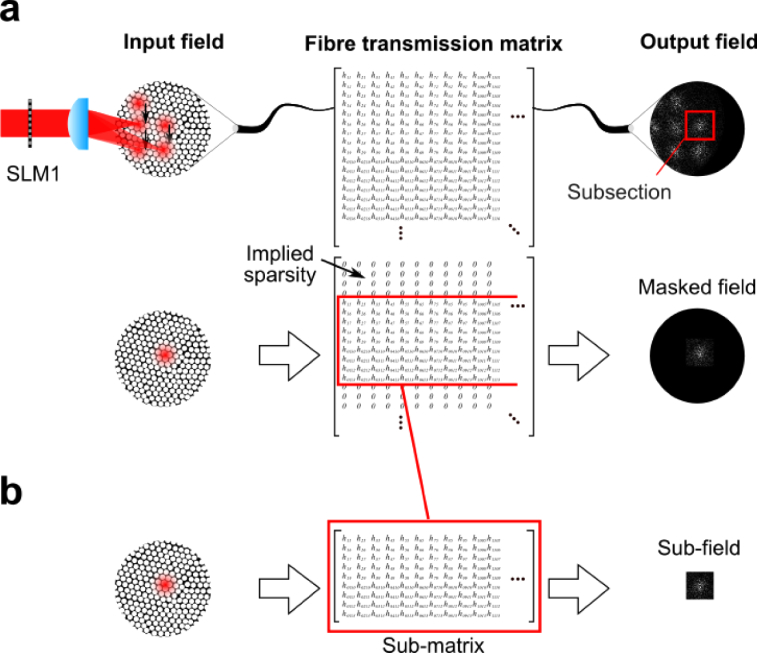
Exploiting the sparse structure of the TM. a) First, the recorded field comprising 12 non-overlapping spots can be split up into 12 separate parts, exploiting the parallelisation of measurements. b) Next, the sparse nature of the TM is further exploited so that only parts of the TM that are known to be non-zero are solved for.

In a pixel basis representation, the resulting fibre bundle transmission matrix is sparse (contains mostly zeroes) and local (the non-zero entries are clustered together). Consequently, a single image containing 12 spots can be split up to produce 12 separate characterisation images. Each image simply uses the relevant subsection of the full image and sets all other pixels to zero (Fig. 4(a)

). This means that there are effectively 12 × 243 = 2916 characterisation images from only 243 actual measurements. Each of the 81 spatial translations effectively provides 12 pixels of resolution at the distal facet (one per spot). Therefore, in total there are 12 × 81 = 972 pixels that can be spatially resolved at the distal facet. This provides a 12-fold improvement over conventional methods and, because the degree of parallelisation is limited only by core-to-core coupling, can be scaled up to larger MCF by simply using more spots – no additional experimental overhead is incurred.

All *P* input field vectors (*P* = 2916 here), $x_{p}$, *p* = 1..*P*, can be concatenated into a matrix, X, as can the associated output field vectors, $y_{p}$, *p* = 1.. *P* to give:

(2) $$X=\left[\begin{array}{llll} x_{1} & x_{2} & \cdots & x_{P} \end{array}\right]\;Y=\left[\begin{array}{cccc} y_{1} & y_{2} & \cdots & y_{P} \end{array}\right]$$

and it follows from Eq. (1) that:

(3) $$X^{T}=Y^{T}{\left(A^{-1}\right)}^{T}$$

The goal is then to solve Eq. (15) for the inverse TM, $A^{-1}$, which is need for image recovery. We can do this by solving the a linear inverse problem for each row:

(4) $$x_{r}^{T}=Y^{T}a_{r}^{T}$$

Here, **x**_*r*_ is a single row of **X** and thus corresponds to a single spatial position across all input calibration fields at the distal facet. Similarly, **a**_*r*_ is a single row of $A^{-1}$ and therefore represents the coupling to the single spatial position represented by **x**_*r*_ from all spatial positions at the proximal facet, represented by columns of **Y**.

We can now further exploit the known sparsity of **A**, and in turn $A^{-1}$, and reconstruct only the parts of $A^{-1}$ where the non-zero elements are located. If the coordinates of the spatial position of $x_{r}$ are (a,b), then the relevant points on the distal facet will be those neighbouring (a,b). We therefore select a set of spatial points on the distal facet (i.e. columns of Y) that are located within a square centred on (a,b) with side length 68.2*μ*m. This length is exactly equal to the spacing between adjacent spots in the array used to characterise the fibre. Equation (4) then becomes:

(5) $$x_{r}^{T}=Y_{sub}^{T}a_{r,sub}^{T}$$

where $Y_{sub}$ is the selected subset of rows of Y and $a_{r,sub}$ is the corresponding subset of columns of $A^{-1}$ (Fig. 4b). The problem is then solved using an L1 regulariser so that the solution to Eq. (5) becomes [32]:

(6) $$\text{min}\;{||a_{r,sub}||}_{1}\;\text{subject to}\;{||x_{r}^{T}-Y_{sub}^{T}a_{r,sub}^{T}||}_{2}<\delta$$

where δ is a parameter that can be adjusted to favour a more or less sparse solution. Equation (6) is solved numerically using a basis pursuit denoising algorithm implemented in the SPGL1 package [33].

### 2.5. Image data recovery and analysis

Having used the method described in Section 2.1 to record raw amplitude and phase images in two polarisations of the proximal MCF facet, the next step is to recover the field at the distal facet of the MCF. A raw amplitude and phase image, $y_{meas}$ is multiplied by the inverse fibre TM **A**^−1^ to give the recovered field at the distal facet, **x**_*dist*_ = **A**^−1^ **y**_*meas*_. This processing was implemented in MATLAB (Mathworks), as are all subsequent processing steps unless otherwise stated.

#### 2.5.1. Correcting for variable working distance

Successful phase imaging makes it possible to correct for defocus arising from the sample being moved away from the distal facet by simulating the propagation of the distal field through free-space using the Fresnel diffraction integral:

(7) $$E\left(x,y,z\right)=\frac{e^{ikz}}{i\lambda z}\iint_{-\infty }^{+\infty }E\left(x\prime ,y\prime ,0\right)e^{\frac{ik}{2z}\left[{\left(x-x\prime \right)}^{2}+{\left(y-y\prime \right)}^{2}\right]}dx\prime \;dy\prime$$

where z is the distance between the desired focal plane and the distal facet, x and y are spatial coordinates in the desired focal plane, and $x^{\prime }$ and $y^{\prime }$ are spatial coordinates in the distal facet plane. Discrete Fourier transform methods were used to compute this integral efficiently following appropriate sampling as previously reported [34].

#### 2.5.2. Extracting polarisation properties

To infer polarimetric properties of a sample, a second and (optional) third set of 11 frames are acquired using different illumination polarisation states. A Jones matrix formalism is then used to model the polarimetric properties of samples. Jones calculus is applicable because the light is temporally and spatially coherent due to the laser diode and the single-mode (or, at worst, few-mode) filtering of the cores respectively, thus depolarisation is negligible [35].

**Fig. 5.**
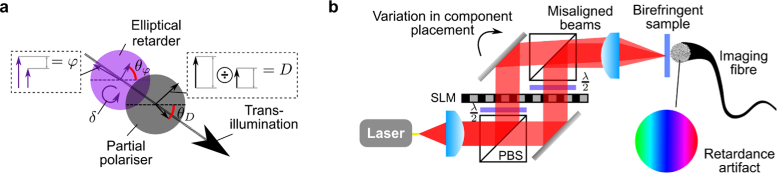
Fitting a model to infer polarimetric parameters of a sample. a) Polarisation model used to factorise Jones matrix at each pixel of sample via Bayesian inference. b) Small misalignments between polarisation arms can result in the beams not being parallel. This is not noticeable if the sample retardance axis orientation ($\theta_{\varphi }$) is aligned with the polarisation axis of the two arms. However, for a general birefringent sample with an arbitrary $\theta_{\varphi }$ the misaligned beams are cross-coupled creating a phase-tilt artefact in the recovered retardance, φ. This artefact can be effectively compensated by re-expressing the problem in a linear polarisation basis at angle ${\hat{\theta }}_{\varphi }$ to the illumination polarisation axis and performing joint inference on neighbouring pixels.

In order to examine the properties of birefringence, diattenuation (also called *linear dichroism*) and circularity, we choose a generalised decomposition of the Jones matrix into an arbitrary elliptical retarder followed by a partial linear polariser (Fig. 5(a)

). The elliptical retarder introduces a phase shift φ (retardance) between the fast and slow optical axes. The retardance axes are oriented at an angle $\theta_{\varphi }$ to the horizontal. There is also a component δ that induces circularity, e.g. representing a chiral molecule. The retarder is followed by a partial linear polariser. The ratio representing the relative power admitted in each of the two axes is D (diattenuation) and the orientation of this diattenuation axis relative to the horizontal is $\theta_{D}$. Each measured point on the sample was modelled using these five parameters.

Having measured a set of n output polarisation vectors from the sample (n=3 here) denoted:

(8) $$V(x,y)=\left[v_{1}(x,y)\cdots v_{n}(x,y)\right]$$

and knowing the corresponding n sample illumination polarisation vectors, denoted:

(9) $$U(x,y)=\left[u_{1}(x,y)\cdots u_{n}(x,y)\right]$$

the 2 × 2 complex Jones matrices J(x,y) representing how the sample maps input polarisation state to output polarisation state at each pixel can be determined through:

(10) V(x,y)=J(x,y)U(x,y)

where J(x,y) is the Jones matrix of a particular pixel at (x,y). We express our decomposition of J(x,y) as:

(11) $$J(x,y)=A_{pol}A_{ret}$$

(12) $$A_{pol}=\left(\begin{array}{cc} \cos\theta_{D} & \sin\theta_{D}\\ -\sin\theta_{D} & \cos\theta_{D} \end{array}\right)\left(\begin{array}{cc} \sqrt{1+D} & 0\\ 0 & \sqrt{1-D} \end{array}\right)\left(\begin{array}{cc} \cos\theta_{D} & -\sin\theta_{D}\\ \sin\theta_{D} & \cos\theta_{D} \end{array}\right)$$

(13) $$A_{ret}=\left(\begin{array}{cc} 1 & 0\\ 0 & e^{i\delta } \end{array}\right)\left(\begin{array}{cc} \cos\theta_{\varphi } & \sin\theta_{\varphi }\\ -\sin\theta_{\varphi } & \cos\theta_{\varphi } \end{array}\right)\left(\begin{array}{cc} e^{i\varphi /2} & 0\\ 0 & e^{-i\varphi /2} \end{array}\right)\cdot \left(\begin{array}{cc} \cos\theta_{\varphi } & -\sin\theta_{\varphi }\\ \sin\theta_{\varphi } & \cos\theta_{\varphi } \end{array}\right)\left(\begin{array}{cc} 1 & 0\\ 0 & e^{-i\delta } \end{array}\right)$$

Bayesian inference using a maximum likelihood optimiser (the STAN package [36]) was applied to each set of n=3 amplitude, phase and polarisation images to extract the individual polarimetric sample properties of: diattenuation (D), also called linear dichroism; diattenuation axis ($\theta_{D}$); retarder circularity (δ); retardance (φ); and retardance axis ($\theta_{\varphi }$). From these equations, it is observed that the inferred parameters are all real numbers limited to the following ranges:

(14) $$\theta_{\varphi }\in (-\pi /2,\pi /2],\;\varphi \in (-\pi ,\pi ],\;\delta \in (-\pi ,\pi ],\;D\in [-1,1],\;\theta_{D}\in (-\pi /2,\pi /2]$$

To fit the model, we evaluate the posterior probability of the parameter set as a function of the data using Bayes’ theorem:

$$\begin{array}{ll} p\;[D,\theta_{D},\varphi ,\theta_{\varphi },\delta |U(x, & y),V(x,y)]\propto \\ & p[V(x,y)=J(D,\theta_{D},\varphi ,\theta_{\varphi },\delta ,x,y)U(x,y)]\cdot p(D,\theta_{D},\varphi ,\theta_{\varphi },\delta ) \end{array}$$

The values in V(x,y) represent measured complex quantities and so are assumed to be independent and drawn from complex Gaussian distributions as:

(15) $$v(x,y)∼CN\;\left[J(D,\theta_{D},\varphi ,\theta_{\varphi },\delta ,x,y)u(x,y),\sigma^{2}I\right]$$

where v(x,y) is a column of V(x,y), u(x,y) is a column of U(x,y), $\sigma^{2}I$ is the covariance matrix, and CN(μ,Σ) is a 2-D complex Gaussian distribution of mean *μ* and covariance Σ. The noise standard deviation, σ, was inferred from the data to have a mean of ∼0.4 and was kept fixed (rather than as a inferred parameter) for subsequent calculations in order to increase speed. We initially consider a broad prior: a 5-dimensional uniform distribution over all the polarisation parameters. In this case, the prior distributions for each individual parameter are independent so that:

(16) $$p(D,\theta_{D},\varphi ,\theta_{\varphi },\delta )=p(D)p(\theta_{D})p(\varphi )p(\theta_{\varphi })p(\delta )$$

In the case of a slightly more restrictive prior, this independence assumption should still be valid to a good approximation and so it used for the rest of the analysis presented here. Priors for parameters representing angles, i.e. circular quantities, are modelled with *von Mises* distributions, with a parameter κ such that 1/κ is analogous to variance [37]. For $\theta_{D}$, $\theta_{\varphi }$ and φ, the κ is set to ∞ providing the broadest possible prior. For δ, a restricted value of κ=1 is used as this is found empirically to reduce over-fitting compared to a uniform prior.

Diattenuation, D, is non-circular so its prior is specified using a truncated Gaussian over its domain [−1,1] with mean 0 and broad variance (=5 here). Again, the broad variance is used to prevent overfitting.

#### 2.5.3. Correcting polarisation misalignment artefacts

Because of unavoidable slight misalignment between the horizontally and vertically polarised arms of the experimental set up (Fig. 1), samples that exhibit large values of retardance, φ, and in which the retardance axes are not aligned with the polarisation axes of the illumination (i.e. ($\theta_{\varphi }\neq 0,\pm \pi /2$), may induce spuriously coupling between the two arms. This produces wavefronts with different tilts in the two polarisation arms, manifesting as a spatially varying retardance artefact (Fig. 5b). This can be compensated by noting that this retardance artefact does not appear when the polarisation axes are aligned with the retardance axes. Therefore, the effect is removed by re-expressing Eq. (10) in a linear polarisation basis at angle ${\hat{\theta }}_{\varphi }$ to the illumination polarisation axes:

(17) $$V(x,y)={J(x,y)|}_{\theta_{\varphi }=0}\left(\begin{array}{cc} \cos{\hat{\theta }}_{\varphi } & \sin{\hat{\theta }}_{\varphi }\\ -\sin{\hat{\theta }}_{\varphi } & \cos{\hat{\theta }}_{\varphi } \end{array}\right)U(x,y)$$

We then apply Bayesian inference in this new, rotated basis with ${\hat{\theta }}_{\varphi }$ taking the place of $\theta_{\varphi }$ as the inferred orientation angle of the retardance axes. To find the ${\hat{\theta }}_{\varphi }$ that minimises the spatial tilt of the retardance we compute the joint probability for a small set of R neighbouring pixels surrounding (x,y). For computational tractability, we employ a technique from variational Bayesian inference that approximates the posterior probability density function as a product of distributions [38]:

$$\begin{array}{ll} & p[D(x_{1},y_{1})\cdots D(x_{R},y_{R}),\theta_{D}(x_{1},y_{1})\cdots ,\varphi (x_{1},y_{1})\cdots ,{\hat{\theta }}_{\varphi }(x_{1},y_{1})\cdots ,\delta (x_{1},y_{1})\cdots \\ & |U(x_{1},y_{1})\cdots ,V(x_{1},y_{1})\cdots ]=\prod_{r=1}^{R}p[D(x_{r},y_{r}),\theta_{D}(x_{r},y_{r}),\varphi (x_{r},y_{r}),{\hat{\theta }}_{\varphi }(x_{r},y_{r}),\delta (x_{r},y_{r})\\ & \;|U(x_{r},y_{r}),V(x_{r},y_{r})] \end{array}$$

where the term inside the product is computed using Eqs. (15) and (17). The probability is maximised when neighbouring pixels have similar conditional distributions for their polarisation parameters. This implicitly biases towards values of ${\hat{\theta }}_{\varphi }$ where the spatial variation of retardance is minimised, i.e. when the means of the retardance distributions across neighbouring pixels are most similar. This approach will provide the best estimate of parameters even if no birefringent tilt artefact is present because the probability distribution of ${\hat{\theta }}_{\varphi }$ tends towards a uniform distribution when φ→0, which is the same behaviour expected for $\theta_{\varphi }$ (i.e. without the correction). Therefore, the correction is applied to all measurements.

The number of adjacent pixels used for the correction is set as R=15. For optimal resolution, this value should be as small as possible because it effectively low-pass filters the image, reducing resolution. On the other hand, larger values of R can correct for smaller tilt artefacts. R=15 was found experimentally to be the smallest value that was sensitive enough to correct the tilt artefact of this system. The outcome of this correction is validated by rotating a half-waveplate in the sample plane (see for example [23]).

Finally, sincewe are decomposing a single matrix into the product of 8 matrices (Eqs. (11)–(13)), each comprising sinusoidal or complex exponential terms, there are degeneracies of this factorisation. Specifically, considering the parameters of Eq. (14), there is an 8-fold ambiguity (or *equivalence class*) for each point in the 5-D parameter space, comprising the following parameter sets:

(18) $$\begin{array}{ccccccc} ( & \theta_{\varphi }, & \varphi , & \delta , & D, & \theta_{D} & )\\ ( & \theta_{\varphi }+\pi /2, & -\varphi , & \delta , & D, & \theta_{D} & )\\ ( & -\theta_{\varphi }, & \varphi , & \delta +\pi , & D, & \theta_{D} & )\\ ( & -\theta_{\varphi }+\pi /2, & -\varphi , & \delta +\pi , & D, & \theta_{D} & )\\ ( & \theta_{\varphi }, & \varphi , & \delta , & -D, & \theta_{D}+\pi /2 & )\\ ( & \theta_{\varphi }+\pi /2, & -\varphi , & \delta , & -D, & \theta_{D}+\pi /2 & )\\ ( & -\theta_{\varphi }, & \varphi , & \delta +\pi , & -D, & \theta_{D}+\pi /2 & )\\ ( & -\theta_{\varphi }+\pi /2, & -\varphi , & \delta +\pi , & -D, & \theta_{D}+\pi /2 & ) \end{array}$$

where parameters are defined to wrap around their ranges, listed in Eq. (14). Without care, these ambiguities could give the misleading impression that polarisation properties exhibit a high degree of variation across an image. To avoid this, we select a point in 5-D parameter space, here we use $\left(\theta_{\varphi }=\pi /4,\varphi =\pi ,\delta =0,D=1,\theta_{D}=\pi /4\right)$, and choose the degenerate solution closest to that point. This ensures the maximum degree of smoothness in the resulting polarisation images. As a result, observed variations can more confidently be classed as structural features rather than artefacts.

### 2.6. Fibrescope performance evaluation.

Imaging was first performed using the simple text pattern hologram (Fig. 3b) to confirm the accuracy of the retrieved TM result. Next, successful phase imaging was confirmed experimentally by performing computational refocusing. A standard 1951 USAF resolution test target (R1DS1N, ThorLabs) was placed at 0 mm working distance from the distal end of the imaging MCF, then displaced in 0.25 mm increments. At each displacement, the lines of known spacing on the target were used to compute the contrast as a function of spatial frequency, i.e. the modulation transfer function (MTF). Threshold values for the MTF used to determine resolution range from 0 to 0.5 in the literature [39] but we noted for all 5 focal distances tested that MTF < 0.3 results in only 2 of the 3 lines being distinguishable by eye. We therefore selected MTF = 0.3 as a threshold. For each displaced target, linear interpolation is used to find the spatial frequency at which MTF = 0.3, which is used to define the image resolution and associated error.

Errors in the amplitude and phase measurements are determined by measuring a known flat sample (a glass slide) and observing the variance of the amplitude and phase over this surface. Such variance is caused by errors in the illumination due to the limitation of hologram generation, and also due to noise in the system. The error in phase between polarisations (i.e. birefringence) is determined by imaging a known, flat waveplate (WPF-2-12.7x12.7-H-850, FOCtek Photonics) and observing the variance of this quantity over the image.

The decomposition of polarisation images into individual polarisation properties (D, $\theta_{D}$, δ, φ, $\theta_{\varphi }$) was evaluated experimentally using two polarisation test targets. The birefringent target (R2L2S1B, Thorlabs) uses the National Bureau of Standards 1963A resolution pattern, with the lines encoded in a highly birefringent liquid crystal polymer. The foreground has the polymer retardance axis oriented at 0° and the background at 45°, testing our ability to recover $\theta_{\varphi }$. To test the recovery of the diattenuation properties D and $\theta_{D}$, we fabricated in-house a bespoke test target using electron beam lithography to produce metallic nanowire gratings with element width and pitch of less than 100 nm [40]. The resulting optical structure is an effective wire-grid polariser and so we encoded a standard 1951 USAF resolution test target with the foreground being a polariser oriented at 0° and the background being a polariser oriented at 90°. Using the lines encoded on these targets, the above approach for measuring resolution using MTF was again applied. Errors in polarimetric properties are determined by propagating errors in amplitude and phase through the Jones matrix model of an elliptical retarder and partial linear polariser of Eq. (11).

## 3. Results

### 3.1. Test pattern imaging

**Fig. 6.**
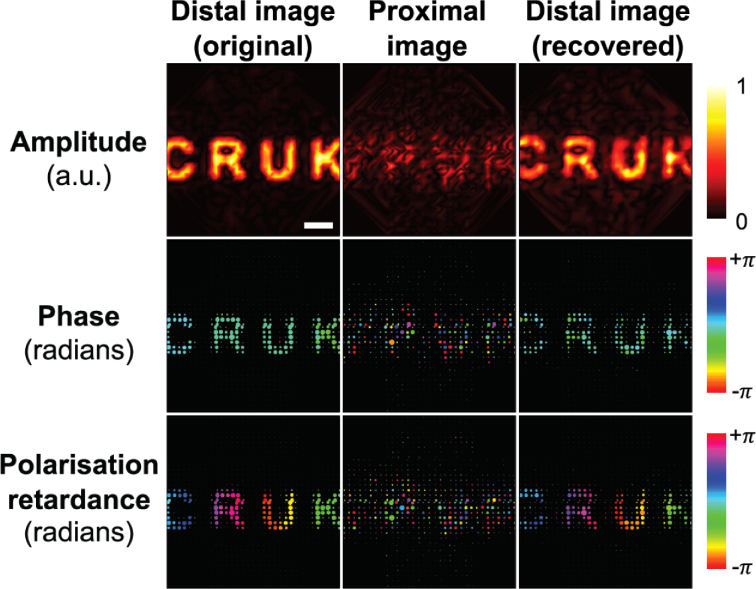
Recovery of the amplitude, phase and polarisation from a test pattern displayed on SLM1 through the MCF by application of the determined transmission matrix. For comparison, the original distal image shown is sub-sampled at the same coordinates used for image reconstruction to represent the best possible recovered image. Bubble area indicates the power recorded within a given point. Image scale bar: 50 *μ*m.

Initially, simple test patterns were displayed on SLM1 for imaging (Fig. 6

), which demonstrated successful recovery of amplitude, phase and polarisation information by multiplying the raw complex image with the inverse of the retrieved TM. The text shape and properties input into the distal facet are visually comparable to those recovered from the measured image of the proximal facet. Next, by imaging known flat samples (a glass slide for phase imaging and a quarter waveplate for polarisation), the phase error was estimated to be 0.3 rad and the polarisation retardance error 0.2 rad. The total acquisition time for an amplitude, phase and polarisation image set is 8.3 s, and the time taken to fully characterise TM of the fibre is 50.8 mins.

### 3.2. Quantitative phase imaging

**Fig. 7.**
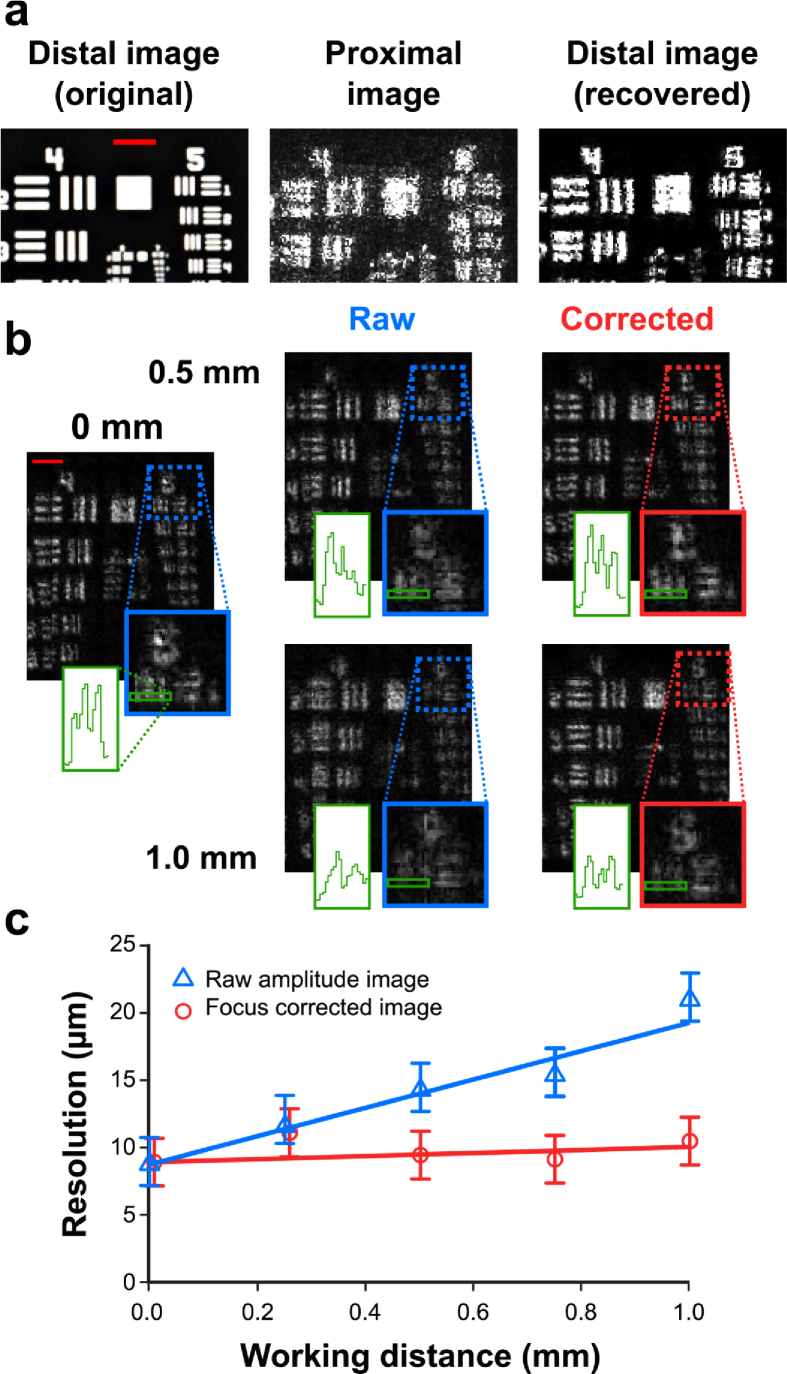
Phase recovery enables computational refocussing. a) Resolution determination with a standard USAF 1951 test chart at 0 mm working distance is 11.0±2.6μm for the proximal facet image and 9.0±2.6μm for the recovered distal facet image. b) As expected, resolution inherently decreases with working distance due to defocus, but using phase information this was corrected over a range of working distances (up to 1.0mm). c) Defocus correction maintains optimal resolution throughout the range of working distances tested (raw amplitude images: y=(10.5±2.7)x+(8.5±1.6), $r^{2}$ = 0.83, p = 0.031; focus corrected images, trend not significant, p = 0.54). Image scale bars: 200*μ*m.

We evaluated recovery of quantitative phase information using a USAF 1951 resolution test chart placed at a range of working distances. Wide-area images of targets larger than the MCF image area (200×200*μ*m at 0 mm working distance) were formed by translating the sample and stitching the sub-images together. Analysis of the modulation transfer function using amplitude-only images of USAF chart line groups shows a lateral resolution of 11.0±2.6*μ*m without TM correction and 9.0±2.6*μ*m with correction at a working distance of 0 mm (Fig. 7(a)

). This is limited by undersampling of the distal facet during TM characterisation: the 12-spot characterisation pattern is translated in 9*μ*m steps, which could be reduced to 4.5*μ*m (the approximate core spacing). In practice, 9*μ*m steps reduced the dataset size for faster computation.

The target was then moved away from the distal facet in steps of 0.25 mm up to 1 mm and the quantitative phase information is utilised to correct defocus of the sample and improve resolution. The resolution halves to 18.0±2.6*μ*m in the uncorrected case, but returns to 10.0±2.6*μ*m with defocus correction applied (Fig. 7b,c). Given the measured fibre NA of ∼0.35, the image area at 1 mm working distance is ∼750×750*μ*m^2^. Variable working distance demonstrates that quantitative phase imaging allows a dynamically reconfigurable resolution and field-of-view. In applications terms, this could enable wide area survey of a given sample, followed by a zoomed analysis at high resolution.

The resolution we achieve is limited by: the low mode density of MCF (i.e. lower effective NA); the undersampling available cores; and the use of wide-field imaging as opposed to confocal scanning. By contrast, high NA MMF imaging systems can achieve submicron resolution [41]. The relatively low NA of our MCF also preferentially rejects out-of-focus light, akin to a relatively large pinhole. Therefore, longer working distances can be achieved with smaller corrective terms before significant degradation occurs.

### 3.3. Polarimetric imaging

Having established accurate retrieval of quantitative phase information, we then implemented a Jones calculus formalism to extract polarimetric properties of samples (model used shown in Fig. 5(a)). Bayesian inference was applied to pairs of input/output polarisation states to estimate these parameters.

**Fig. 8.**
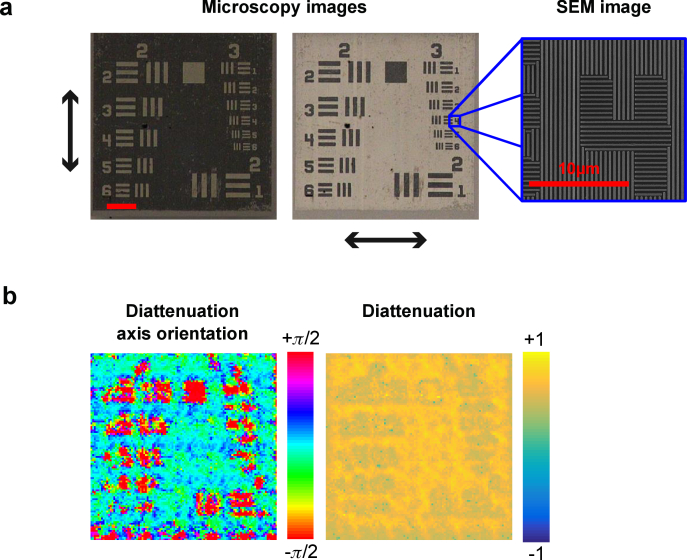
Polarisation-resolved properties can be extracted: Diattenuation. a) A custom diattenuation target encoded test patterns in the diattenuation axis orientation, as illustrated by optical microscopy (image scale bar: 100*μ*m) and scanning electron microscopy (SEM, image scale bar: 10 *μ*m). Arrows on microscopy images indicate polarisation directions of vertical (left) and horizontal (right). b) Test target features appear strongly in the diattenuation axis orientation, with negligible impact on the diattenuation itself.

**Fig. 9.**
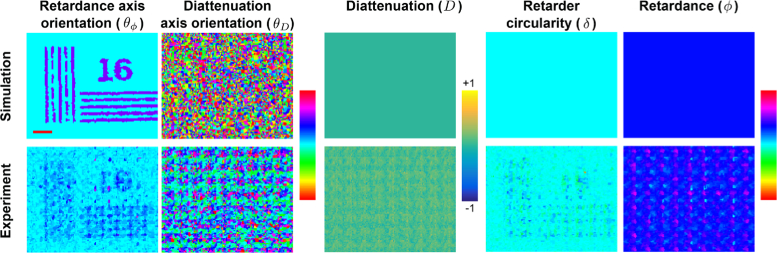
Polarisation-resolved properties can be extracted: Birefringence. A birefringent USAF test target with patterns encoded in the retardance optic axis orientation shows clear signals in this polarisation parameter, with negligible impact on other parameters (image scale bar: 100*μ*m).

Two polarisation test targets were used to validate polarimetric imaging. First, a polarising resolution target fabricated in-house using nanostructured metallic wire-grid polarisers (Fig. 8(a)

) was imaged. Contrast is encoded in $\theta_{D}$ and this is observed experimentally (Fig. 8b). Second, a birefringent resolution target was imaged. The birefringent target creates contrast via a 45° difference between the retardance axis in the foreground and background, i.e. structure is encoded only in $\theta_{\varphi }$. This is observed in experimental measurements (Fig. 9

) and there is good agreement between simulation and experiment in this and across all other properties. In this latter case the diattenuation, D, approaches zero and so the diattenuation axis, $\theta_{D}$, being the argument of a zero-vector, is not well-defined. Therefore, the recovery algorithm renders this uncertainty as random noise for $\theta_{D}$.

The spatial resolution of the $\theta_{\varphi }$ and $\theta_{D}$ images was determined to be 36.0±10.4*μ* m. The reduction in resolution compared to phase imaging is a result of averaging (joint inference) between neighbouring pixels to remove misalignment artefacts (Section 2.5.3). Error propagation from raw phase and amplitude data is used to compute errors of each polarimetric parameter: ΔD=0.14, $\Delta \theta_{D}=0.28$rad, Δδ=0.12rad, Δφ=0.26rad, $\Delta \theta_{\varphi }=0.13$rad.

## 4. Discussion

Parallelised TM characterisation has been shown here to enable imaging of amplitude, quantitative phase and resolved polarimetric properties through an MCF with potential for flexible operation. While some previous work has indirectly used phase and/or polarisation information to recover amplitude-only images from MMFs [7], our holographic fibrescope is the first to directly retrieve wide-field en-face images of these properties together with amplitude information.

Our holographic fibrescope records high resolution (9.0±2.6*μ*m amplitude, phase; 36.0±10.4*μ*m polarimetric) images at working distances up to 1 mm with field-of-view up to 750×750*μ*m^2^. This limit arises from experimental space constraints but could in principle be extended to working distances of 1-2 cm and fields-of-view of ∼1×1 cm^2^. The ability to adaptively change working distance without distal optics is the first key strength of our approach. Quantitative phase is measured with an error of 0.3 rad while polarimetric properties are inferred with errors across the 5 parameters of <10%. The precision and resolution obtained in the quantitative phase and resolved polarimetric properties are the second key strength of our approach, being in principle sufficient to resolve small, localised modulations. This is partly due to the robust Bayesian inference approach used to extract polarimetric parameters that can compensate for misalignment artefacts and noise.

In contrast to previous work using MMFs, the known near-diagonal structure of the MCF TM was used here to implement a novel parallelised architecture that offers a 12-fold speed increase compared to characterisation of a MMF with a dense TM. The reported parallelised architecture is scalable such that no extra experimental time is required for higher resolution MCFs with a greater number of cores. This is especially important when considering that, in real usage, the fibre will be bent or otherwise perturbed, requiring regular re-characterisation of the TM. An equivalent scheme could be implemented with MMFs by changing the optical field representation basis from the point-wise basis used here to a basis matching the spatial eigenmodes of the fibre e.g. Laguerre-Gauss modes. However, the strong axial symmetry of such bases necessitates very precise fibre alignment, reducing robustness to movement and temperature changes typically encountered in clinical environments. Further, large pre-computed hologram libraries are required to create these modes [22], whereas the modes used here are generated *ad-hoc* by applying a phase tilt to a single pre-computed hologram. A key advantage of our approach is that the point-wise basis does not require alignment with an axis and inherently benefits from a near-diagonal TM because of the pixellated structure of the MCF.

Although these results are promising, the speed of operation should be increased for future applications of the approach. Current speeds for amplitude, phase and polarisation imaging (8.3 seconds per image), polarimetric imaging (3×8.3=24.9 seconds), and TM characterisation (50.8 minutes) are limited by camera and SLM frame rates. We estimate that it would be possible to reduce imaging time to <0.05 s for real-time operation and characterisation time to <1 min, using strategies such as: high-speed imaging using a higher frame rate camera or photodiode-based compressive imaging; and replacing LCoS SLMs with fast digital micromirror devices that can achieve up to 100-fold increase in speed for wavefront shaping [42]. Using an appropriately designed phase-mask in place of the current Fresnel lens, it would be possible to use only 2 images for phase retrieval and 3 for polarimetry, offering a further 2-fold speed up. TM computation and image recovery could be reduced to <1 min and <0.1 s respectively by replacing the current iterative approach (∼1000 Fourier transforms required taking 67s per through-focus stack) with transport-of-intensity equation methods (two Fourier transforms and one derivative required, taking <0.2s per through-focus stack) [25]. Furthermore, frameworks that avoid the need for explicit TM reconstruction and directly reconstruct the image data have already been reported [43,44]. Finally, the presented approach uses an SLM to achieve plane-wave illumination of the sample in several polarisation states, which may not be practical for realistic deployments in confined spaces, e.g. an endoscope. Plane-wave llumination could instead be achieved via single-mode polarisation-maintaining fibres [29] or by integrating a miniaturised rotating waveplate with a light source at the distal end of the fibre [45]. Alternatively, if the fibre TM is known structured illumination (in space and polarisation) can be used which further improves resolution [46,47].

## 5. Conclusion

In conclusion, we present a multi-core fibre approach to produce full-field en-face images of amplitude, quantitative phase and resolved polarimetric properties. Using this proof-of-concept holographic fibrescope, we demonstrate that these additional optical properties enable dynamic refocusing at working distances up to 1 mm and provide spatial resolution of polarisation information across the field of view.
